# The ecology and plasticity of fish skin and gill microbiomes: seeking what matters in health and disease

**DOI:** 10.1093/femsre/fuaf027

**Published:** 2025-06-19

**Authors:** Jamie McMurtrie, Ashley G Bell, Joanne Cable, Ben Temperton, Charles R Tyler

**Affiliations:** Biosciences, Faculty of Health and Life Sciences, University of Exeter, Exeter, Devon EX4 4QD, United Kingdom; Sustainable Aquaculture Futures, University of Exeter, Exeter, Devon EX4 4QD, United Kingdom; Biosciences, Faculty of Health and Life Sciences, University of Exeter, Exeter, Devon EX4 4QD, United Kingdom; Sustainable Aquaculture Futures, University of Exeter, Exeter, Devon EX4 4QD, United Kingdom; School of Biosciences, Cardiff University, Cardiff CF10 3AX, United Kingdom; Biosciences, Faculty of Health and Life Sciences, University of Exeter, Exeter, Devon EX4 4QD, United Kingdom; Biosciences, Faculty of Health and Life Sciences, University of Exeter, Exeter, Devon EX4 4QD, United Kingdom; Sustainable Aquaculture Futures, University of Exeter, Exeter, Devon EX4 4QD, United Kingdom

**Keywords:** stressor, dysbiosis, aquaculture, environment, mucous, immune, animal health

## Abstract

The microbiomes of skin and gill mucosal surfaces are critical components in fish health and homeostasis by competitively excluding pathogens, secreting beneficial compounds, and priming the immune system. Disruption of these microbiomes can compromise their capacity for disease resilience and maintaining host homeostasis. However, the extent and nature of microbiome disruption required to impact fish health negatively remains poorly understood. This review examines how various stressors influence the community composition and functionality of fish skin and gill microbiomes, and the subsequent effects on fish health. Our findings highlight that the impact of stressors on skin and gill microbiomes may differ for different body sites and are highly context-dependent, influenced by a complex interplay of host-specific factors, stressor characteristics, and environmental conditions. By evaluating current knowledge on the genesis and homeostasis of these microbiomes, we highlight a strong influence of environmental factors especially on skin and gill microbiomes compared with fish gut microbiomes, which appear to be more closely regulated by the host’s homeostatic and immunological systems. This review emphasizes the importance of understanding the ecology and plasticity of fish skin and gill microbiomes to identify critical thresholds for microbiome shifts that impact fish health and disease resilience.

## Teaser

This review investigates microbiome responses to stressors in the aquatic environment and the nature of microbiome disruptions required to impact fish health negatively. Microbiomes are resilient, requiring functionality assessments in future research to understand interplay between microbiomes and fish health.

## Introduction

Early biological investigations of disease processes focused on identifying pathogens as causative agents. However, more recent studies have shown that nonpathogenic organisms can affect the disease process and form part of the diverse microbial communities associated with maintaining host health (Belkaid and Hand 2014, Thomas et al. 2017, Zheng et al. 2020). These communities, known as the microbiota, are comprised of bacteria, archaea, microeukaryotes, fungi, and protists, with the microbiome encompassing all microbiota and their associated products, including metabolites, mobile genetic elements, and viruses (Berg et al. 2020). Microbiomes form symbiotic relationships with animal (and plant) hosts, whereby the host provides a favourable colonization environment, and commensal microbes synthesize key micronutrients (such as vitamin B12) and initiate immune system priming (Belkaid and Hand 2014, Kelly and Salinas 2017, Legrand et al. 2018). In the absence of a microbiome, the host has a greater disease susceptibility, as demonstrated in gnotobiotic fish (Pérez-Pascual et al. 2021).

Most host-associated microbiome studies have focused on terrestrial animals due to their significance in human health and livestock production. In contrast, relatively little research has been carried out on the microbiomes of fish, which comprise nearly 50% of all vertebrate species and are crucial for global food security and aquatic ecosystem function (Food and Agriculture Organization of the United Nations (FAO) 2020, IUCN Red List 2022). There are strong similarities in gut microbiomes of terrestrial vertebrates and fish, but microbiomes of the lung and skin mucous membranes of terrestrial animals differ more widely from their tissue equivalents—gill and skin, of fish (Hsia et al. 2013, Schröder and Bosch 2016). These differences likely stem from the direct interaction of these surfaces with air in the case of mammals and water in the case of fish. As such, these different environments will differ in their influence on the genesis, retention, and function of host-associated microbiomes (Callewaert et al. 2020). Aquatic environments host diverse and dynamic microbial communities (which facilitate more effective disease transmission) than air that has relatively sparse microbiota (Gupta et al. 2017).

Studies have shown microbiomes on external surfaces of fish (skin, fins, gills, and nares) are comprised of diverse microbes derived from the surrounding environment, and influenced by host physiology and environmental factors, including water physicochemistry (Horsley 1977, Arias et al. 2013, Lowrey et al. 2015, Chiarello et al. 2018). These dynamic microbial communities respond to internal and external factors, exhibiting variations even among conspecifics (Boutin et al. 2013, Tarnecki et al. 2019, Uren Webster et al. 2020b). Microbiome plasticity aids in buffering against changes in microbial population structure, thereby resisting functional alterations in response to stressors and providing resilience against disease onset. Defining a healthy community, however, is challenging as interindividual and intersurface variations and temporal fluctuations in these communities are natural. However, exposure to stressors, which surpass a microbiome’s buffering capacity can disrupt the host–microbe symbiotic relationship and reduce host fitness (Carlson et al. 2015, Uren Webster et al. 2021). When the protective effects of a microbiome are diminished, the host organism is at greater disease susceptibility.

Disease is a major cause of fish mortality in aquaculture, costing the global industry an estimated USD 6 billion annually and hindering the industry’s expansion and sustainability (Akazawa et al. 2014, Stentiford et al. 2017). Infectious disease events in fish are often preceded by stressors that increase the likelihood of infection and disease due to various interacting components of fish mucosal health (Segner et al. 2012, Masud 2020). Impaired skin and gill immune responses often occur when fish are stocked at inappropriate densities, tending to result in greater mortality rates when subjected to pathogen challenge (Ellison et al. 2018, 2020). Various stressors, such as hypoxia, can also alter the synthesis of adhesins and antimicrobial peptides, which are important in pathogen virulence and defence (Pérez-Sánchez et al. 2017, Sanahuja et al. 2019). Studying mucosal health in response to stressors, particularly the relationship between microbiomes and the host, can therefore provide key insights into host fitness, health, disease susceptibility, and microbiome dysbiosis.

Dysbiosis is a concept for understanding how microbiomes respond to stressors, the subsequent impact on their functional capacities, and host susceptibility to disease. Dysbiosis is characterized by disruption in the microbiome causing it to transition to a state that may facilitate disease and detrimental health outcomes (DeGruttola et al. 2016). Host physiology is also impacted, where dysbiosis may alter mucus production (Navabi et al. 2013), interfere with membrane trafficking processes (Weber and Faris 2018) and/or trigger inflammation (Borton et al. 2017), which in turn disrupts a microbiome’s community structure and function. It is important to recognize that due to considerable interindividual variation in the microbiome, there is no single healthy, dysbiotic, or diseased state. However, there are hallmarks of dysbiosis that include loss of commensals (natural residents that contribute positively to host and microbiome function), enrichment of pathobionts (commensals capable of contributing to disease pathology under appropriate conditions), and loss of microbial diversity (Petersen and Round 2014, DeGruttola et al. 2016, Levy et al. 2017). Understanding how fish microbiomes react and change in response to external and internal factors is fundamental to establishing their role in animal health and defining commonalities in dysbiotic prognosis.

Various reviews have described fish microbiomes and their interactions with the immune system (Kelly and Salinas 2017, Yu et al. 2021). Recently, two reviews on fish skin microbiomes have provided descriptions of skin microbial composition and recommendations for the standardization of microbiome analysis (Gomez and Primm 2021, Wang et al. 2023). Recent research studies have also investigated fish microbiome shifts in response to specific stressors (Debnath et al. 2023, Gómez de la Torre Canny et al. 2023, Hamilton et al. 2023, Rosado et al. 2023, Sánchez-Cueto et al. 2023). However, little is known about what constitutes a shift in fish microbiomes to a nonhealthy or dysbiotic state and how this affects fish fitness and disease progression. Here, we address the biological and microbial processes governing fish skin and gill microbiome composition, how these microbiomes respond to stressors, the impact these perturbations may have on fish health, and present recommendations for approaches to better assess fish microbiomes and their functional states.

## Processes governing the composition and natural assembly of fish skin and gill microbiomes

### Healthy microbiome(s)

Healthy microbiomes comprise a diverse community of commensals that prime the immune system (Levy et al. 2016, Murdoch and Rawls 2019) and defend against pathogenic colonization by competing for resources and secreting antimicrobial compounds (de Kamada et al. 2013, Bruijn et al. 2018). Microbiota colonization affects host physiology in mucosal and nonmucosal tissues (Massaquoi et al. 2023) as demonstrated in gnotobiotic models of early life-stage fish. For instance, gnotobiotic Atlantic salmon (*Salmo salar*) fry have a reduced skin mucous layer thickness, which is reversed upon recolonization of naive skin mucosa by microbiota, including *Pseudomonas* and Comamonadaceae species (Gómez de la Torre et al. 2023). Similarly, colonization of gnotobiotic fish by commensals has also been shown to protect against infection with *Flavobacterium columnare* in rainbow trout (*Oncorhynchus mykiss*) (Pérez-Pascual et al. 2021) and *Vibrio harvei* in seabass (*Dicentrarchus labrax*) (Schaeck et al. 2016).

**Figure 1.**
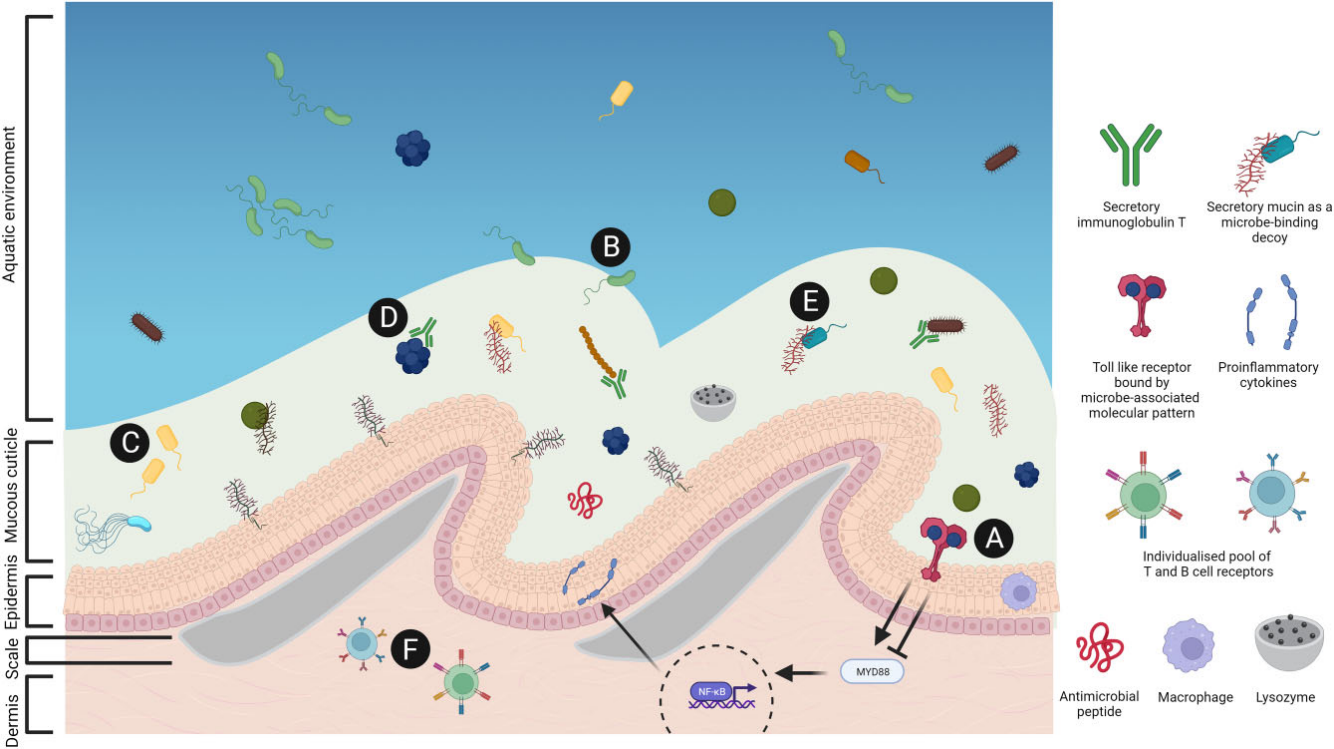
Host-immune factors influencing the microbiota in the skin mucosal microbiome. (A) TLRs recognize microbe-associated molecular patterns, activating proinflammatory signalling cascades (MyD88) and transcription factors (NF-κB) to prime the immune system whilst also preventing excessive inflammation through negative feedback mechanisms. (B) Mucosal microbiomes may harbour transient taxa from the aquatic environment, potentially colonizing if mucosal conditions change. (C) Microbes adapted to mucosal niche conditions successfully colonize the host microbiome under niche appropriation theory, regardless of rarity in the surrounding environment. (D) Secretory IgT binds commensals and pathogens in skin mucous, preventing migration into subepithelial structures. (E) Secretory mucins bind and confine microbes to the mucosal layer, influenced by variable glycosylation patterns. (F) Somatic mutations of B- and T-cell receptors during development lead to the creation of unique sets of immune receptors for each individual, shaping microbiota selection. Other innate immune components that can contribute to shaping mucosal microbiome compositions include antimicrobial peptides, macrophages, and lysozymes. Created with BioRender.

While the role of commensal microbiota in priming the host immune system is well-characterized in mammalian systems (Zheng et al. 2020), their role in fish immune systems remains poorly understood. In fish, immune signalling can be host-derived, such as microbiota-induced serum amyloid A mediating neutrophil migratory behaviours (Kanther et al. 2014) or microbiota-derived, such as the secretion of the antiinflammatory factor AimA by *Aeromonas* commensals (Rolig et al. 2018). As fish constantly encounter a wide variety of planktonic microbiota, their immune system must effectively differentiate between commensal and pathogenic microbiota to avoid excessive inflammatory responses. Commensal colonization primes the fish’s innate immune system by recognizing microbial-associated molecular patterns through toll-like receptors (TLRs) (Fig. 1A). Recognition triggers the proinflammatory MyD88 signalling cascade, activating transcription factors such as NF-κB (Galindo-Villegas et al. 2012), which is crucial for regulating numerous innate immune genes (Kanther et al. 2011). Additionally, the TLR2-MyD88 pathway provides negative feedback to commensal colonization in gnotobiotic zebrafish, by preventing disproportionate inflammation under normal conditions (Koch et al. 2018). This balance between pro- and antiinflammatory signals is important for successful host–microbiota symbiosis.

Beyond immune modulation, fish skin and gill microbiota have important host-specific functions that contribute to key physiological processes. For instance, toxic waste products excreted at the gill are removed by the gill microbiota through ammonia oxidation and denitrification (van Kessel et al. 2016). Furthermore, commensals excrete host-beneficial compounds, including antimicrobial metabolites (*Pesudoaltermonas* spp.; Offret et al. 2016), bioactive metabolites (*Vibrionaceae* spp.; Mansson et al. 2011), and vitamin B12, exclusively synthesized by prokaryotes and essential for animal life (*Cetobacterium somerae*; Tsuchiya et al. 2008).

### Composition of fish skin and gill microbiomes

Fish skin and gill microbiome compositions are host-specific (Chiarello et al. 2018, Pratte et al. 2018) and influenced by environmental factors, such as water salinity, pH, and divalent cations (Lokesh and Kiron 2016). These microbial communities have been described elsewhere (Llewellyn et al. 2014, Legrand et al. 2020b), and specifically for fish gill (Chen et al. 2023a) and skin microbiomes (Gomez and Primm 2021, Debnath et al. 2023, Wang et al. 2023). Common findings across studies are the dominance of the bacterial phylum Pseudomonadota (formerly Proteobacteria), particularly from the class Gammaproteobacteria. However, core microbiota compositions can vary between different fish taxa when assessed at the genus level (Larsen et al. 2013, Boutin et al. 2014, 2015, Schmidt et al. 2015, Carda-Diéguez et al. 2017, Chiarello et al. 2018, Pratte et al. 2018, Sylvain et al. 2019). Initial investigations into microbiota functionality have used shotgun metagenomic sequencing; the skin microbiome of eel (*Anguilla anguilla*) reveals enrichment in genes related to biofilm formation, quorum sensing, competition, adherence, and immune system evasion, functional capacities that are likely required for successful bacterial colonization of the fish skin (Carda-Diéguez et al. 2017).

Swab sampling of fish external mucosal surfaces recovers both autochthonous microbiota (resident taxa permanently colonizing the mucosal surface) and allochthonous microbiota (taxa that transiently inhabit the mucosal surface and are generally free-living, not permanently colonizing it). While transient taxa may not permanently establish themselves, they may still contribute significantly to the community by interacting with resident microbes and the host immune system, altering nutrient availability and increasing microbial competition. However, the functional impact of transient taxa on host health and the broader microbiome remains unclear. Under conducive conditions, transient taxa may transition to become permanent residents. This shift may lead to new microbiome ‘states’, where the balance between resident and new colonizing taxa alters microbiome functionality with unknown implications for host health and disease resilience.

### Fish skin and gill microbiome assembly theories

Two theories of microbial community assembly include niche appropriation and neutral theory. Niche appropriation suggests that competitive interactions between species dictate assembly, as each species occupies distinct ecological niches based on unique traits (Hutchinson 1959). Rare but well-adapted microbes can outcompete more abundant but less specialized individuals. Alternatively, neutral theory suggests that assembly reflects the surrounding environmental community, as all species are equally competitive and stochastic (random) processes drive microbiome structure (Hubbell 1979, Chisholm et al. 2004). Importantly, host microbiomes have specific conditions that limit colonization to a subset of bacteria, preventing unsuitable environmental microbes from establishing, regardless of assembly theory (Fig. 1B).

Niche appropriation theory appears particularly relevant for fish microbiomes, as the microbiome on the same mucosal surface is more similar between conspecifics than between different mucosal sites within the same individual (Sylvain et al. 2016, Reinhart et al. 2019, Minich et al. 2020). Niche appropriation theory is particularly supported in a study by Chiarello et al. (2018) as only 3% of the variation in skin microbial composition of coral reef fish could be explained by the environmental reef habitat, compared to explaining 20% variation in planktonic community composition. Thus, specific taxa that are best adapted to conditions of the skin mucosal surface are retained from the water column. Further evidence of this can be seen by rare aquatic taxa becoming enriched in fish microbiomes, as seen in the case of *Vibrio*, which comprises around 1.7% of water microbiota but 26% of fish skin microbiota (Schmidt et al. 2015). This suggests that specific immune or physiological factors on fish mucosal surfaces, along with microbial adaptations, contribute to the selection and retention of microbes in the fish microbiome (Chiarello et al. 2018, Dash et al. 2018).

Neutral theory also holds merit in explaining fish microbiome assembly. For example, stochastic models best explain the initial colonization of the skin microbiome in tambaqui (*Colossoma macropomum*), where skin microbiome differences were observed between fish in different tanks, but not between those in the same tank. However, these differences diminished over time (Sylvain et al. 2016). The host mucosal surface likely acts as a habitat filter for the stochastic colonization of taxa from the surrounding environment, leading to the formation of an initial unstable microbiome community composition. Over time, niche appropriation enables better-adapted rare taxa to proliferate in these niches, determining a new and stable microbial community composition (Schmidt et al. 2015) (Fig. 1C). Collectively, these processes contribute to the unique and variable microbiome compositions seen in individual fish.

### Environmental influences on fish skin and gill microbiomes assembly

The environment plays a crucial role in shaping fish skin and gill microbiomes. For example, in outdoor aquaculture, tilapia skin microbiomes have been shown to cluster by culture pond (McMurtrie et al. 2022). Similarly, wild Amazonian freshwater fish species (flag cichlid *Mesonauta festivus* and black piranha *Serrasalmus rhombeus*) show habitat-driven differences in skin (Sylvain et al. 2019) and gill (Sylvain et al. 2023) microbiomes, likely driven by different physicochemical conditions (Sylvain et al. 2019).

Translocation studies offer compelling evidence of environmental influence on the external fish microbiome (skin and gill). For instance, Atlantic salmon fry translocated from the wild to artificial hatchery conditions undergo a near-complete skin and gill microbial turnover, which becomes indistinguishable from their original habitats while alpha diversity remains unchanged (Uren Webster et al. 2020b). Despite developing healthy microbiomes based on their environment, certain taxa such as *Rickettsiaceae* spp. were sustained after translocation indicating that early-life colonization influences the core microbiome (Uren Webster et al. 2020b).

Aquaculture systems can also affect microbiome composition. In Atlantic salmon, differences in skin and gill microbiome beta diversity were found between flow-through and recirculating aquaculture systems (Minich, Poore et al. 2020). Similarly, yellowtail kingfish (*Seriola lalandi*) reared in different aquaculture systems (flowthrough, BioGil RAS, or moving bed bioreactor RAS) showed differences in alpha and beta diversity of the gill microbiome but not the skin microbiome (Minich et al. 2021).

Social environments similarly can impact fish microbiomes, as seen in Caribbean broadstripe cleaning gobies (*Elacatinus prochilos*) that were found to have differences in the alpha and beta diversities of their skin microbiome when residing in ecotypes as individuals versus when in social groupings (Xavier et al. 2019). Similarly, clownfish (*Amphiprion clarkii*) housed with sea anemones experienced transient changes in their skin microbiome composition, including enrichment of *Rubritalea* sp. as they underwent fish–anemone mutualism (Pratte et al. 2018), even without physical contact (Émie et al. 2021).

These observations highlight the substantial influence of the environment on skin and gill microbiomes, with differing responses occurring at these different tissue surfaces (Minich et al. 2021, Lorgen-Ritchie et al. 2022, 2022). Divergent fish microbiome compositions potentially reflect plasticity—a hallmark of a healthy and functionally stable community, as demonstrated in human systems (Huttenhower et al. 2012). However, it remains unclear if the observed differences across different environments are associated with microbiome fitness and resilience. Pathogen or other physicochemical stressor challenge studies are needed to determine the robustness of the different microbiomes in protecting against adverse health outcomes.

### Host and immune processes contributing to microbiome assembly

The contribution of environmental and host factors to fish microbiome assembly varies for the different mucosal surfaces. In coral reef fish, gill microbiomes are more similar to the gill microbiome of other fish, compared to the gut microbiomes of the same fish, indicating body site-driven microbiome shaping (Pratte et al. 2018). In particular, genotype is crucial in shaping fish skin and gill microbiomes (Chiarello et al. 2015, 2018, Rosado et al. 2019a, Minich et al. 2022). For instance, in brook charr (*Salvelinus fontinalis*), host genotype has been shown to dictate the abundance of dominant commensals such as *Methylobacterium* (Boutin et al. 2014). While host-specific influences on fish skin microbiomes can be identified, phylosymbiosis patterns are not always obvious, as microbiome composition does not appear to align consistently with host taxonomic distance (Doane et al. 2020, Bell et al. 2024). However, a recent study suggests significant (although weak) phylosymbiosis in skin and gill microbiomes across 101 marine fish species (Minich et al. 2022).

The immune system also plays a vital role in regulating skin and gill microbial communities. Mucosal-associated lymphoid tissues (MALT), composed of myeloid and lymphoid cells, work with innate and adaptive immune processes to differentiate between commensals and pathogens to mediate microbiome compositions (Salinas 2015, Yu et al. 2021). The multifaceted nature of the immune system adds complexity in understanding host immune response roles in microbiota colonization and dysbiosis. Illustrating this, infection of rainbow trout by the ciliated parasite *Ichthyophthirius multifiliis* resulted in upregulation of immune complement-related genes, proinflammatory cytokines, T cell-related cytokines, and antimicrobial peptides accompanied by a decrease in skin Proteobacteria (specifically *Acinetobacter, Shewanella*, and *Pseudomonas*) and an increase in the prevalence of pathobionts (specifically *Flavobacterium*) (Zhang et al. 2018).

Secretory immunoglobulins, particularly secretory immunoglobulin T (sIgT), are vital for maintaining mucosal surface homeostasis and defending against pathogens (Fig. 1D). sIgT coats the majority of bacterial microbiota on fish skin and gills (Xu et al. 2013, 2016) (Fig. 1D). Transient depletion of sIgT in adult rainbow trout leads to invasion of bacteria into gill epithelium and extensive dysbiosis of the gill microbiome. This dysbiosis is characterized by the loss of key commensals and proliferation of pathobionts, which is reversed upon sIgT recovery to basal levels, indicating its role in microbiota stability (Xu et al. 2020).

Mucins, similar to slgT, help limit microbe penetration to mucosal layers (Fig. 1E). Their glycosylation patterns influence microbiome selection and pathogen control by binding bacterial lectins (Arike and Hansson 2016, Sheng and Hasnain 2022), trapping microbes in microbe–mucin conjugates (Linden et al. 2008, Benktander et al. 2019) (Fig. 1E). In rainbow trout, skin mucins enriched with short-chain glycans prevent microbial adherence to epithelial cells while gill-secreted mucins bind to pathogens aiding in their clearance (Thomsson et al. 2022). As such, variations in mucin glycosylation across host species may drive differences in microbiome composition.

Gut immune processes share parallels with the skin and gill immunity, including MALT structure and immune components (Xu et al. 2013, Yu et al. 2021). Insights from the gut may therefore inform of immune influences over the skin and gill microbiomes. For example, macrophages are crucial in microbiota selection, as macrophage deficient zebrafish lose core gut commensals such as *Cetobacterium* spp. (Earley et al. 2018). Similarly, knockout of proinflammatory cytokine IL-17A/F1 in medaka (*Oryzias latipes*) alters innate humoral components expression, leading to decreased gut microbiome richness, altered community structure, and increased *Plesiomonas* genera abundance (Okamura et al. 2020, 2021). IL-17A/F is highly expressed in various mucosal tissues, including the skin and gills, further highlighting potential immune-mediated microbiome regulation of the skin and gills (Zhou et al. 2021).

The adaptive immune system also acts as an ecological filter to shape microbial communities. During development, somatic mutation of B- and T-cell receptors creates a personalized pool of receptors to influence microbiota selection (Weinstein et al. 2009) (Fig. 1F). This is demonstrated by wildtype zebrafish exhibiting greater gut beta diversity dissimilarity compared to *rag1*- zebrafish mutants, which lack adaptive immune components (B- and T-cell receptors). Therefore, a functional adaptive immune system filters microbiota and structures host–microbiota assembly (Stagaman et al. 2017). Together, the complex interplay of innate and adaptive immune processes suggests how fish, even in early development stages, shape a unique microbiome at their mucosal surfaces (Fig. 1).

### Fish skin and gill microbiome responses to environmental stressors

Fish skin and gill microbiomes can undergo major compositional shifts in response to environmental stressors, ranging from natural events, such as changes in water salinity that occur as salmon migrate between rivers and the sea (Glaser and Kiecolt-Glaser 2005) and to adverse events like disease, which result in dysbiosis (Mohammed and Arias 2015, Carlson et al. 2017, Legrand et al. 2018, 2020). Stressors can also impact planktonic microbial communities that interact with fish skin and gill microbiomes (Schmidt et al. 2015) and/or induce physiological and immunological changes in host mucosal surfaces, favouring colonization of microorganisms adapted to new mucosal conditions (Meng et al. 2021). Disruption of microbial community interactions may lead to a loss of microbiological function (Cheaib et al. 2021), which can manifest within several hours. Here, we critically assess the effects of physical (Table 1), biological (Table 2) and chemical (Table 3) stressors on fish skin and gill microbiomes. These assessments, however, are limited to studies performing 16S rRNA metabarcoding with comparisons available against a control group, or a timeseries where natural disease outbreaks have been tracked. Furthermore, reported alterations in taxa abundance need to be substantiated statistically against relevant controls, and not simply based on descriptive observations of apparent increases or decreases. Our analysis reveals little consistency in gill and skin microbial composition, richness, or diversity in response to different stressors.

**Table 1.** Physical stressors of fish skin and gill microbiomes. Studies included performed high throughput amplicon sequencing and applied statistical tests to assess significance. NC = no statistically significant change (α = 0.05). NR = not reported. Five differentially abundant taxa with the greatest effect size are displayed per study.

Stressor	Mucous surface	Species	Stressor strength	Stressor duration	Alpha diversity	Beta diversity	Differential increase in genera abundance	Differential decrease in genera abundance	Reference
Temperature	Skin	Chum salmon *Oncorhynchus keta*	8°C, 13°C, 18°C	14 days	NR	*R* ^2^ = NR, *P* < .0009	*NR*	*NR*	Ghosh et al. (2022)
Temperature	Skin	Greater amberjack *Seriola dumerili*	29°C	90 days	NC	*R* ^2^ = 0.28., *P* = .002	*Psychrobacter, Pseduoalteromonas, Paracoccus, Planococcus, Chryseomicrobium*	*Polaribacter, Nautella, SAR92, Pseudophaeobacter, Lentibacter*	Sánchez-Cueto et al. (2023)
	Gill				NC	*R* ^2^ = 0.24., *P* = .002	*Psychrobacter, Paracoccus, Planococcus, Chryseomicrobium, Pseduoalteromonas*	*Polaribacter, Nautella, SAR92, Pseudophaeobacter, NS3a marine group*	
	Skin		33°C		NC	*R* ^2^ = 0.23., *P* = .002	*Psychrobacter, Pseduoalteromonas, Paracoccus, Planococcus, Polaribacter*	*Polaribacter, Nautella, Pseudophaeobacter, SAR92, Lentibacter*	
	Gill				NC	*R* ^2^ = 0.22, *P* = .002	*Psychrobacter, Exiguobacterium, Paracoccus, Planococcus, Chryseomicrobium*	*Polaribacter, Nautella, SAR92, NS3a marine group, Pseudophaeobacter*	
Temperature	Skin	Pacific chub mackerel *Scomber japonicus*	12°C–24°C	1-year timeseries	↑	NC	*NR*	*NR*	Minich, Petrus et al. (2020)
	Gill				NR	NC	*NR*	*NR*	
Chlorophyll a	Skin		0–5 µg/l		↓	NC	*NR*	*NR*	
	Gill				↓	NC	*NR*	*NR*	
Temperature (acute cold stress to eggs)	Skin	Atlantic salmon *S. salar*	0.2°C and air exposed	5 min per stressor	NS	*R* ^2^ = NR, *P* = .001	*Acinetobacter, Aeromonas, Pseudorhodobacter, Mycoplasma, Gemmatimonas*	*Pseudomonas, Janthinobacterium, Staphylococcus, Mycoplasma, Methylobacterium*	Uren Webster et al. (2021)
Salinity (saltwater transition)	Skin	Atlantic salmon *S. salar*	35 PSU	4 weeks	↑	*R* ^2^ = NR, *P* = NR	*Psychromonas, Marinomonas, Pseudoalteromonas, Acrobacter, Polaribacter*	*Sphingomonas, Acinetobacter, Polynucleobacter, Pseudomonas, Stenotrophomonas*	Lokesh and Kiron (2016)
Salinity (saltwater transition)	Skin	Arctic Char *Salvelinus alpinus*	880–3450 µS/cm	N/A	↓	*R* ^2^ = NR, *P* < .001	*NR*	*NR*	Hamilton et al. (2019)
Salinity (freshwater transition)	Gill	Marine medaka *Oryzias melastigma*	Seawater to fresh	14 days	↓	NR	*Pseudomonas, Polynucleobacter, Oleibacter, Shewanella*	*Tenacibaculum, Haloferula, Pseudomonas, Salinivibrio, Ruegeria*	Lai et al. (2022)
Salinity	Skin	Black molly *Poecilia sphenops*	5 ppt	30 days	NR	*R* ^2^ = 0.09, *P* < .13	*Cetobacterium, Shewanella, Aeromonas, Vibrio*	*NR*	Schmidt et al. (2015)
			18 ppt			*R* ^2^ = 0.88, *P* < .0006	*Vibrio, Shewanella*	*Cetobacterium, Aeromomnas*	
			30 ppt			*R* ^2^ = 0.96, *P* < .0002	*Vibrio, Shewanella*	*Cetobacterium, Aeromomnas*	
pH	Skin	Tambaqui *C. macropomum*	pH 4	2 weeks	NC	*R* ^2^ = 0.57, *P* < .05	*Undibacterium*	*Flavobacterium*	Sylvain et al. (2016)
Netting	Skin	Atlantic salmon *S. salar*	NA	30 s	NC	*R* ^2^ = NR, *P* < .01	*NR*	*NR*	Minniti et al. (2017)
Confinement stress	Skin	Atlantic salmon *S. salar*	5.6 fish/l	1 h daily, 2 weeks	NC	*R* ^2^ = NR, *P* = .15	*NC*	*NC*	Uren Webster et al. (2020a)
Confinement stress and hypoxia	Skin	Brook charr *S. fontinalis*	8 fish/l and dO_2_ 3 mg/l	5 min	NC	NR	*NR*	*Methylobacterium, Sphingomonas*	Boutin et al. (2013)
Chronic air exposure	Skin	Gilthead seabream *Sparus aurata*	2x a week, 4 weeks	1 min	↑	*R* ^2^ = 0.49., *P* < .001	*Pseudoalteromonas, Marinagarivorans*	*Acinetobacter, NS3a_marine_group, Pseudomonas*	Cámara-Ruiz et al. (2022)
Chronic environmental stress	Skin	Atlantic salmon *S. salar*	Housing lacking shelter	1057-degree days	NC	*R* ^2^ = NR, *P* = .001	*Streptococcus*	*NC*	Uren Webster et al. (2021)

**Table 2.** Biological stressors of fish skin and gill microbiomes. Studies included performed high throughput amplicon sequencing and applied statistical tests to significant effects. NC = no statistically significant change (α = 0.05). NR = not reported. Five differentially abundant taxa with the greatest effect size are displayed per study.

Stressor	Mucous surface	Species	Stressor strength	Stressor duration	Alpha diversity	Beta diversity	Differential increase in genera abundance	Differential decrease in genera abundance	Reference
*Neoparamoeba perurans* Amoebic gill disease	Gill	Atlantic salmon *S. salar*	100 *N. perurans*/l	1 h	↓	*R* ^2^ = NR, *P* < .05	*Tenacibaculum, Propionibacterium, Pseudoalteromonas, Mesorhizobium*	*Arcobacter, Aestuariicella*	Slinger et al. (2020)
*N. perurans*, Amoebic gill disease	Gill	Atlantic salmon *S. salar*	Natural outbreak	4 months	↑	NR	*NR*	*NR*	Birlanga et al. (2022)
*Lepeophtheirus salmonis*, sea lice	Skin	Atlantic salmon *S. salar*	8 *L. salmonis /* l	1 h	↑	*R* ^2^ = 0.35, *P* < .001	*Rhizobiales, NS10_marine_group*	*Arthrobacter*	Llewellyn et al. (2017)
*Ichthyophthirius multifilii*, ich	Skin	Rainbow trout *O. mykiss*	5000 theronts per fish	24 h	↑	*R* ^2^ = NR, *P* < .05	*Actinobacter, Bdellovibrio, Clostridiales, Flavobacterium*	*Acinetobacter, Pseudomonas*	Zhang et al. (2018)
*Sparicotyle chrysophrii*	Gill	Gilthead seabream *Sparus aurata*	Water effluent from infected fish	42 days	↓	*R* ^2^ = 0.58., *P* < .001	*Branchiomonas, Ichthyocystis, Polaribacter*	*Staphylococcus, Shewanella, Escherichia*	Toxqui-Rodríguez et al. (2024)
Spring viremia of carp virus	Skin	Carp *Cyprinus carpio*	I.P. inject 1 × 10^7^ pfu	4 days	NC	NR	*Turicibacter*	*Sphingomonas, Sphingobacterium*	Meng et al. (2021)
	Gill				↓	NR	*Aquabacterium, Azospirllum*	*Acinetobacter*	
Salmonid alphavirus	Skin	Atlantic salmon *S. salar*	7 or 139 TCID50 SAV3/l	6 h	NC	*R* ^2^ = NR, *P* < .05	*NC*	*NC*	Reid et al. (2017)
Salmonid alphavirus	Skin	Atlantic salmon *S. salar*	48 TCID50 SAV3/l	6 h	↓	*R* ^2^ = NR, *P* < .05	*NR*	*NR*	Brown et al. (2021)
	Gill				NC	NC	*NR*	*NR*	
Infectious hematopoietic necrosis virus	Gill	Rainbow trout *O. mykiss*	10^7^ TCID50 IHNV/l	2 h	↓	NR	*Achromobacter, Paracoccus, Peanarthrobacter*	*Rhodococcus, Deinococcus, Reyranella, Aurantimicrobacterium*	Tongsri et al. (2023)
Infectious hematopoietic necrosis virus	Skin	Rainbow trout *O. mykiss*	1 × 10^9^ pfu/ml	2 h	↑	*R* ^2^ = 0.58, *P* < .001	*Rhodococcus, Vibrio, Acinetobacter, Flavobacterium, Uruburella*	*NR*	Zhan et al. (2022)
Gut enteritis	Skin	Yellowtail kingfish *S. lalandi*	Natural outbreak, early enteritis	NA	↓	*R* ^2^ = 0.65, *P* < .0001	*Loktanella, Marivita, Planktomarina, Simplicispira, Litoricola*	*Ascidiaceihabitans, Roseovarius, Ferrovum, Glaceicola, Synechococcus*	Legrand et al. (2018)
	Gills				NC	*R* ^2^ = 0.58, *P* < .0001	*Loktanella, Marivita, Simplicispira, NS5 marine group, Microcella*	*Ascidiaceihabitans, Roseovarius, Glaceicola, Psychrobacter, Salimicrobium*	
*Vibrio harveyi*	Skin	European seabass *D. labrax*	Natural outbreak	NA	↑	*R* ^2^ = 0.52, *P* = .002	*Vibrio*	*Rubritalea*	Cámara-Ruiz et al. (2021)
*Photobacterium damselae*	Skin	European sea bass *D. labrax*	Natural outbreak, mortality induced	NA	↓	*R* ^2^ = 0.4, *P* = .06	*NR*	*NR*	Rosado et al. (2019b)
	Gill				↑	*R* ^2^ = 0.3, *P* = .004	*NR*	*NR*	
*P. damselae* subsp. *piscicida* and *Vibrio harveyi*	Skin	European sea bass *D. labrax*	Natural outbreak, mortality induced	NA	↑	*R* ^2^ = 0.3, *P* = .02	*NR*	*NR*	Rosado et al. (2022)
*P. damselae* subsp. *piscicida*	Skin	Gilthead Seabream *Sparus aurata*	Natural outbreak, mortality induced	NA	NC	NC	*NR*	*NR*	Rosado et al. (2023)
	Gill				NC	*R* ^2^ = 0.7, *P* = .02	*NR*	*NR*	
*Aeromonas hydrohpilia*	Skin	Striped Catfish *Pangasianodon hypophthalmus*	10^6^ CFU/ml	5 days	NC	*R* ^2^ = 0.28, *P* = .002	*Vibrio, Corynebacterium, Paracoccus, Brevundimonas, Escherichia*	*NR*	Chen et al. (2022)
*Aeromonas salmonicida*	Skin	Rainbow trout *O. mykiss*	10^6^ CFU/ml	1 h	NC	*R* ^2^ = 0.25, *P* = .038	*NR*	*NR*	Redivo et al. (2023)
	Gills				NC	*R* ^2^ = 0.25., *P* < .02	*NR*	*NR*	
Bacteriophage (*P. damselae* subsp *damselae*)	Whole larval fish	Longfin yellowtail *Seriola rivoliana*	1.41 × 10^10^ PFU/ml	12 days	NC	NR	*NC*	*NC*	Veyrand‐Quirós et al. (2021)
Probiotics (*P. inhibens S4Sm* and *B. pumilus RI06-95Sm*)	Skin	Black molly *Poecilia sphenops*	1 × 10^5^ CFU/ml	5 days	NC	NC	*NC*	*NC*	Schmidt et al. (2017)
*Bdellovibrio*	Gill	Crucian carp *Carassius auratus*	NR	60 days	NC	NR	*NR*	*NR*	Zhang et al. (2023)
Prebiotics (mannans, beta glucans, fatty acids)	Skin	Atlantic salmon *S. salar*	0.5, 1.0, 2.0 g/kg	12 weeks	↓ (1 g/kg); NC (0.5 and 2 g/kg)	NC	*Bacillus, Granulicatella, Mycetocola, Paraperlucidibaca*	*Alcanivorax, Halomonas, Paracoccus, Chryseobacterium, Idiomarnia*	Baumgärtner et al. (2022)
Insect meal diet	Skin	Rainbow trout *O. mykiss*	100% fish meal replace	22 weeks	NC	NC	*NC*	*Deefgea*	Terova et al. (2021)
Invertebrate enriched diet	Skin	Atlantic salmon *S. salar*	5 g invertebrate mix daily	6 weeks	↑	NR	*Deefgea, Flavobacterium, Aeromonas, Chryseobacterium, Undibacterium*	*MD3-55, Plesiomonas, Psychrobacter, Streptococcus, Lawsonella*	Uren Webster et al. (2020b)
*Aphanomyces* (Epizootic ulcerative syndrome)	Skin	Hybrid snakehead (*Channa maculata* x *C. argus*)	Natural outbreak	NA	NR	*R* ^2^ = NR, *P* < .001	*Anaerovorax, Anaerosinus, Dorea, and Clostridium*	*Arthrobacter, Bacillus, Lactococcus, Achromobacter, Pseudomonas*	Li et al. (2019)

**Table 3.** Chemical stressors of fish skin and gill microbiomes. Studies included performed high throughput amplicon sequencing and applied to assess significance. NC = no statistically significant change (α = 0.05). NR = not reported. Five differentially abundant taxa with the greatest effect size are displayed per study.

Stressor	Mucous surface	Species	Stressor strength	Stressor duration	Alpha diversity	Beta diversity	Differential increase in genera abundance	Differential decrease in genera abundance	Reference
Flumequine	Skin	European seabass *D. labrax*	35 mg/kg	7 days	↑	*R* ^2^ = NR, *P* = .006	*NR*	*NR*	Rosado et al. (2022)
Oxytetracycline	Skin	European seabass *D. labrax*	35 mg/kg	8 days	NC	*R* ^2^ = 0.1, *P* = .01	*NR*	*NR*	Rosado et al. (2019a)
	Gill				NC	*R* ^2^ = 0.1, *P* = .04	*NR*	*NR*	
Oxytetracycline	Skin	Gilthead Seabream *Sparus aurata*	150 mg/kg	10 days	NC	NC	*NR*	*NR*	Rosado et al. (2023)
	Gill				↓	*R* ^2^ = 0.5, *P* = .03	*NR*	*NR*	
Oxytetracycline	Gill	Atlantic salmon *S. salar*	79 mg/kg	10 days	NC	NR	*NR*	*NR*	Slinger et al. (2021a)
Florfenicol			10 mg/kg		NC	NR	*NR*	*NR*	
Oxytetracycline, Metronidazole, erythromycin mix	Skin	Yellowtail kingfish *S. lalandi*	OTC 200 mg/kg; MET 50 mg/kg; ERY 50 mg/kg	Oral gavage single dose	NC	*R* ^2^ = NR, *P* < .001	*Tenacibaculum, Oleiphilus, Glaciecola, Paraglaciecola*	*NR*	Legrand et al. (2020)
Tetracycline	Gill	Marine medaka *Oryzais melastigma*	43.6 µg/l	30 days	NR	NC	*Qipenqyuania, Pseudarthrobacter*	*Vibrio, Ruegeria*	Liao et al. (2023)
Streptomycin sulfate	Skin	Black molly *Poecilia sphenops*	200 µg/ml	13 days	NC	NC	*NC*	*NC*	Schmidt et al. (2017)
Cadium chloride	Skin	Yellow perch *Perca flavescens*	9 µg/l	3 months	↑	*R* ^2^ = NR, *P* = .003	*Direction of change not reported: Emticicia, Flavobacterium, Pseudorhodobacter, Shinella, Sphaerotilus*		Cheaib et al. (2021)
Copper sulfate	Gill	Yellow catfish *Pelteobagrus fulvidraco*	0.7 mg/l	7 days	↓	NR	*Plesiomonas, Polynucleobacter, Curvibacter, Aurantimicrobium*	*Sphingopyxis, Paucibacter, Legionella*	Zhou et al. (2023)
Glycophospohate herbicide	Gill	Rainbow trout *O. mykiss*	123 ng/l daily	6 months	NC	NC	*Flavobacterium, Polynucleobacter, Rhodoferax, Candidatus Branchiomonas*	*Limnohabitans*	Bellec et al. (2022)
Crude oil	Skin	Red Snapper *Lutjanus campechanus*	1 ppm	28 days	NC	NC	*Lewinella, Algoriphagus, Arcobacter, Vibrio*	*Marinobacter, Shewanella, Halomonas, Photobacterium*	Tarnecki et al. (2022)
Polystyrene microbeads 38 µm	Gill	Discus fish *Symphysodon aequifasciatus*	20 and 200 µg/l	28 days	↑	*R* ^2^ = 0.93, *P* < .001	*NR*	*Rombutsia, Cetobacterium*	Huang et al. (2022)
	Skin			28 days	NC	*R* ^2^ = 0.74, *P* < .001	*NR*	*NR*	
Polystyrene microbeads 10 µm	Gill	Marine medaka *Oryzais melastigma*	10 µg/l	30 days	NR	NC	*Acidipila, Cavicella, Marvinbryantia*	*Vibrio, Ruegeria*	Liao et al. (2023)

### Physical stressors

Water physicochemistry plays a major role in shaping aquatic microbial communities (Bolaños et al. 2022) and fish skin and gill microbiomes. Water temperature changes can affect skin microbiome beta diversity, although effects on alpha diversity vary among species (Minich et al. 2020, Uren Webster et al. 2021, Ghosh et al. 2022, Sánchez-Cueto et al. 2023). In greater amberjack (*Seriola dumerili*), shifts in the gill microbiome occurred without changes in water microbiomes indicating a host-driven response to water temperature change (Sánchez-Cueto et al. 2023). Salinity transitions, particularly in diadromous fish, can result in substantial changes in skin and gill microbiomes (Schmidt et al. 2015, Lokesh and Kiron 2016, Hamilton et al. 2019, Lai et al. 2022, 2023). However, small salinity changes appear to have minimal impact on microbiome diversity as shown in Pacific chub mackerel (*Scomber japonicus*) (Minich et al. 2020), and various coral reef fish species (Chiarello et al. 2018). In black molly (*Poecilia sphenops*), salinity shifts >5 ppt were required to drive any substantial change in the skin microbiome beta diversity (Schmidt et al. 2015). While water temperature and salinity are well-studied, less is known about the effects of pH and dissolved oxygen (Table 1). In the case of acidic conditions, (pH 4 versus pH 7) an enrichment of *Undibacterium* and depletion of *Flavobacterium* occured in the skin microbiome of tambaqui (Sylvain et al. 2016). It should be recognized that many of the described changes in the skin and gill microbiomes represent their plasticity as a homeostatic response to support microbiome functionality, rather than any dysbiotic state that may render them more susceptible to disease or a lowered health status.

Mechanical damage to the skin and gill surfaces from netting, high stocking densities, or contact with environmental substrates may affect the surface mucosal microbiomes (Table 1). Repeated netting of Atlantic salmon has been shown to increase the skin surface microbiome alpha diversity and alter the abundance of prominent genera (Minniti et al. 2017). Similarly, mechanical stress (through repeated vortexing) of mosquito fish (*Gambusia affinis*) led to altered skin bacterial function (enzymatic activities), though this was recovered after 7 days, albeit through a different taxonomic composition (Brumlow et al. 2019). Confinement-related stress in brook charr (*S. fontinalis*) (Boutin et al. 2013) and Atlantic salmon (Uren Webster et al. 2020a) reduced key skin microbiome commensals including *Methylobacterium* and *Sphingomonas* spp.

### Biological stressors

#### Disease-causing agents

Opportunistic pathogens, even at low abundances, can exploit disruptions in host mucosal physiology, worsening dysbiosis and potentially initiating disease states (Bass et al. 2019). In Atlantic salmon, amoebic gill disease (AGD), caused by *Neoparamoeba perurans*, disrupts the gill microbiome through lesions leading to epithelial cell proliferation (Munday et al. 2001) and excessive mucus secretion (Marcos-López et al. 2018). AGD infection also alters mucin glycosylation, impacting bacterial adhesion (Marcos-López et al. 2017, Benktander et al. 2020), reducing immune enzymatic activities (Marcos-López et al. 2017) and immune gene expression (Botwright et al. 2021). AGD-related changes in gill physiology correspond with shifts in the gill microbiome, characterized by an increased abundance of *Tenacibaculum* (Slinger et al. 2020, 2021b, Birlanga 2022). However, evidence shows contrasting direction and significance of changes to gill alpha diversity during AGD (Slinger et al. 2020, Birlanga 2022). Similar shifts in skin and gill microbiomes have been observed with other ectoparasites, including sea lice (*Lepeophtheirus salmonis*) (Llewellyn et al. 2017), ciliates (*I. multifiliis*) (Zhang et al. 2018), *Chilodonella hexasticha* (Bastos Gomes et al. 2019), and monogeneans (*Sparicotyle chrysophrii)* (Toxqui-Rodríguez et al. 2024) (Table 2).

Viral infections can also disrupt fish skin and gill microbiomes by triggering widespread immune responses. In rainbow trout infected with infectious hematopoietic necrosis virus, antibacterial responses in the skin and gill altered both alpha and beta diversity and enriched putative pathobionts (Zhan et al. 2022, Tongsri et al. 2023). Carp infected with spring viremia of carp virus showed increased expression of innate immune genes IL-1β, NOD1, TNF, and hepcidin, reductions in gill alpha diversity and depletion of various commensals, such as *Sphingomonas* in the skin and *Acinetobacter* in the gill (Meng et al. 2021). Viral-induced microbiome disruption in fish mucosal surfaces (Table 2) may be partially mediated by nonspecific immune responses, with tissue damage facilitating opportunistic taxa proliferation.

Host systemic infections can lead to microbiome disruption at distant mucosal body sites. In yellowtail kingfish with suspected gut enteritis, beta diversity changes were observed in both skin and gill microbiomes, with skin alpha diversity also decreasing. Specific taxa in the skin and gill such as *Loktanella, Marivita*, and *Simplicispira* increased while *Ascidiaceihabitans, Roseovarius*, and *Glaceicola* decreased (Legrand et al. 2018), likely mediated in the skin microbiome by changes in immune expression (Legrand et al. 2020a).

Bacterial infections often lead to an increase in pathogenic taxa, such as *Tenacibaculum* and *Photobacterium*, which can cause disease directly or exacerbate existing disease conditions, as observed in skin ulcers of Atlantic salmon (Karlsen et al. 2017). Broader microbiome disruption, including the loss of key skin commensals like *Rubritalea*, was observed during an outbreak of *Vibrio harveyi* in European seabass (Cámara-Ruiz et al. 2021). The infection dose can also influence microbiome change as seen in striped catfish (*Pangasianodon hypophthalmus*) exposed to *Aeromonas hydrophilia*, where differences in skin beta diversity occurred only when the infection dose was >10^5^ CFU/ml (Chen et al. 2022). It is also the case that responses to infection may vary between the skin and gill tissues. This is reported for infections of *Photobacterium damselae* in European seabass, where there was reduced skin alpha diversity but increased gill alpha diversity (Rosado et al. 2019a, 2022, Cámara-Ruiz et al. 2021). These findings underscore the variability in the microbiome response to disease.

#### Health treatments

Bacteriophages, probiotics, and dietary components are widely utilized in aquaculture to promote health and mitigate disease (Table 2), though their effects on the skin and gill microbiomes have been little studied. Bacteriophages are gaining attention for disease treatment due to their narrow bacterial host range. In longfin yellowtail (*Seriola rivoliana*), bacteriophages have been used to effectively reduce *P. damselae* subsp. *damselae* abundance and provide disease protection (Veyrand‐Quirós et al. 2020, 2021). However, phage treatment may also induce broader microbial disruptions, as seen in studies on larval fish microbiomes (Veyrand‐Quirós et al. 2021) and the gut microbiome of Atlantic salmon (Donati et al. 2022). This could occur through the lysis of phage hosts, allowing alternative taxa to fill vacated niches within the microbiome.

Probiotics, living organisms used to enhance host health may also influence the host’s microbiome. For example, in black molly and brook charr, probiotic strains of *Phaeobacter inhibens* S4Sm and *Bacillus pumilus* RI06-95Sm colonize the skin mucosa and protect against *Vibrio anguillarum* colonization without significant alterations of the skin microbial composition (Boutin et al. 2013). Similarly, in Nile tilapia (*Oreochromis niloticus*), *Bacillus cereus*, and *Alcaligenes faecalis* have been shown to confer a protective health effect without disruption to the skin and gill microbiomes (Wang et al. 2020a). However, predatory probiotics like *Bdellovibrio* sp., used to combat *Aeromonas hydrophila* in crucian carp (*Carassius gibelio*) were found to alter the gill microbiome, enriching it with taxa belonging to the Proteobacteria phylum (Zhang et al. 2023). Such probiotics may influence microbial networks to favour certain taxa but, probiotics offering transient synergism generally do not cause broader disruptions in fish skin or gill microbiomes (Table 2).

Although many studies have investigated alternative fish feeds to promote growth or enhance disease resilience, their effects on the skin microbiomes remain largely uncharacterized. Most research, including studies on pufferfish (*Takifugu obscurus*) (Yang et al. 2007), yellow grouper (*Epinephelus awoora*) (Feng et al. 2010), Atlantic salmon (Landeira-Dabarca et al. 2013, Schmidt et al. 2016), and rainbow trout (*O. mykiss*) (Terova et al. 2021), report no major effects on skin bacterial diversity. However, Atlantic salmon fed a mixture of invertebrates, in addition to a standard commercial feed, exhibited increased skin alpha diversity, with enrichment of *Aeromonas* and *Flavobacterium* (Uren Webster et al. 2020b). Similarly, prebiotically fed Atlantic salmon showed an enrichment of *Bacillus* and depletion of *Chryseobacterium*, an emerging salmonid pathogen (Baumgärtner et al. 2022). Whilst commercial diets generally are reported to have minimal effects on fish skin and gill microbial diversity, plant-based diets are reported to alter mucin and antimicrobial peptide expression patterns in the skin and gill of Atlantic salmon (Sørensen et al. 2021). Thus some feed additives may induce alterations to mucosal physiology and result in effects on skin and gill microbiomes (Table 2).

### Antibiotics and other chemicals

Antibiotics are widely used in aquaculture for disease treatment and prevention (Rosado et al. 2022, Thornber et al. 2022), but can disrupt microbiomes. They are furthermore common pollutants in waterways (Wilkinson et al. 2022). During disease outbreaks, antibiotics generally have negligible impacts on skin and gill alpha diversity, but they cause temporary changes in beta diversity. These changes in the microbiome generally return to a state similar to the initial healthy, or predisease, state within 1–2 weeks postexposure (Rosado et al. 2019a, 2023, Legrand et al. 2020, Slinger et al. 2021a). As a consequence of antibiotic exposure a fish skin microbiome can become enriched with pathobionts. For instance, in yellowtail kingfish antibiotic treatment enriched the skin with *Tenacibaculum* and other taxa responsible for ulcerative disease (Legrand et al. 2020). However, skin and gill tissues can respond differently to antibiotics. As an example, in gilthead seabream (*Sparus aurata*) treated with oxytetracycline, alpha diversity decreased and beta diversity shifted in the gill microbiome but not the skin microbiome (Rosado et al. 2023). Antibiotics also induce significant shifts in healthy fish skin microbiomes. This is evidenced by studies on mosquitofish (*G. affinis*) treated with rifampicin, where there was a transient loss of culturable bacteria in the skin and enrichment of specific taxa such as *Myroides, Vibrio, Pseudomonas*, and *Mitsuaria*, accompanied by biochemical functional changes (Carlson et al. 2017). The route of antibiotic application is also likely to influence the microbiome response. Illustrating this for the gut microbiome, administration of enrofloxacin to tilapia via injection, oral dosing, or via the water, resulted in significantly differing gut microbiome responses (Chen et al. 2023b). However, such studies have not been conducted to assess for effects on skin or gill microbiomes, where the impact of exposure routes are likely to differ from those seen in the gut.

Other chemical pollutants in surface waters have been shown to impact fish skin and gill microbiomes (Table 3). Examples, of this include exposure to heavy metals. Illustrating this, cadmium chloride exposure (9 ppb) increased skin alpha diversity and caused a more segregated and modular community network structure in the skin microbiome in yellow perch (*Perca flavescens*) (Cheaib et al. 2020, 2021). Similarly, environmentally relevant exposures of glyphosate herbicide reduced gill microbiome connectivity between functional modules in rainbow trout (Bellec et al. 2022). Surprisingly, crude oil exposure has been shown to have minimal effects on fish skin and gill microbiomes (Table 3). After the 2010 Deepwater Horizon oil spill, Gulf killifish (*Fundulus grandis*) (Larsen et al. 2015) and red snapper (*Lutjanus campechanus*) exposed to crude oil mimicking the Deepwater Horizon incident, showed no significant differences in skin microbiome composition, although some taxa exhibited differential abundance (Tarnecki et al. 2022).

Microplastic pollution also affects fish skin and gill microbiomes, with reports of remodelling in outer-facing mucosal microbiomes (Table 3). Discus fish (*Symphysodon aequifasciatus*) exposed to polystyrene microplastics at environmentally relevant concentrations showed substantial differences in beta diversity compositions skin and gill microbiomes (Huang et al. 2022). However, in marine medaka (*Oryzais melastigma*) gill microbiomes, a similar exposure had no significant effect (Liao et al. 2023). However, when the marine medaka were exposed to a combination of the polystyrene microplastics and the antibiotic tetracycline, there was an enhanced antibiotic effect on the skin microbiome. Thus, underscoring the need to assess the combined effects of multiple stressors on fish microbiomes as will occur in natural systems.

## Changes in skin and gill microbiomes relevant for health

**Figure 2.**
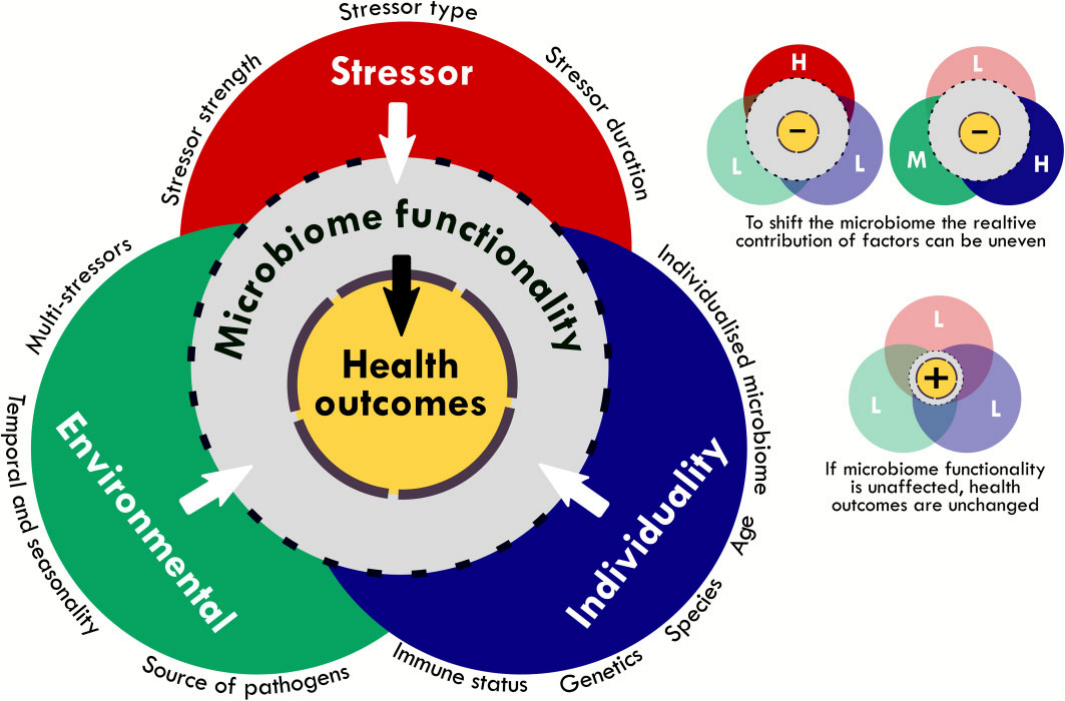
Microbiome shifts impacting animal health. Left-hand side (LHS): stressor-induced microbiome shifts depend on three factors: (1) stressor characteristics—duration and intensity must be sufficient to cause change. (2) Host individuality—each host’s unique microbiome affects its susceptibility and resilience to shifts, influenced by factors such as age, species, and immune status. (3) Environmental conditions—factors such as temperature, pH, and diurnal/seasonal patterns can impose selective pressures on mucosal physiology. The aquatic environment also acts as a reservoir for potential pathogens that exploit microbiome shifts. Right-hand side (RHS): the impact of stressors, the host, and/or environmental conditions may vary depending on the relative strength of the stressor/environmental condition and susceptibility of the host (indicated as low, medium, or high). Even a low strength stressor can alter microbiome functionality if the host is highly susceptible, or the environment amplifies the effect. Health outcomes decline only if microbiome functionality is disrupted.

As we illustrate above, alterations in fish skin and gill microbiomes can occur due to a variety of factors, but what matters is whether these alterations functionally impact the fish’s health. (Fig. 2). To date, no single microbiome compositional or diversity shift has been consistently linked to a specific stressor, with variability in the response to a stressor also occurring between conspecifics (Minich et al. 2020, 2022, Bell et al. 2024). As such, the relevance of microbiome alterations to animal health is highly context-dependent, and influenced by many factors (Fig. 2).

The relative contributions of stressors to shift a microbiome is mediated by stressor characteristics, the individuality of a host, and environmental conditions, but individually or collectively these factors have to be of a sufficient magnitude to disrupt microbiome functionality. Understanding these functional consequences is crucial to determining the impact of stressor-induced microbiome shifts on fish health. Microbiomes are capable of buffering against stressor action, for example through functional redundancy (Doane et al. 2023), and the capability of individuals to do so helps explain variation in the impact of stressor responses on health between individuals within a given fish population.

### Microbiome alterations impacting disease resilience

**Figure 3.**
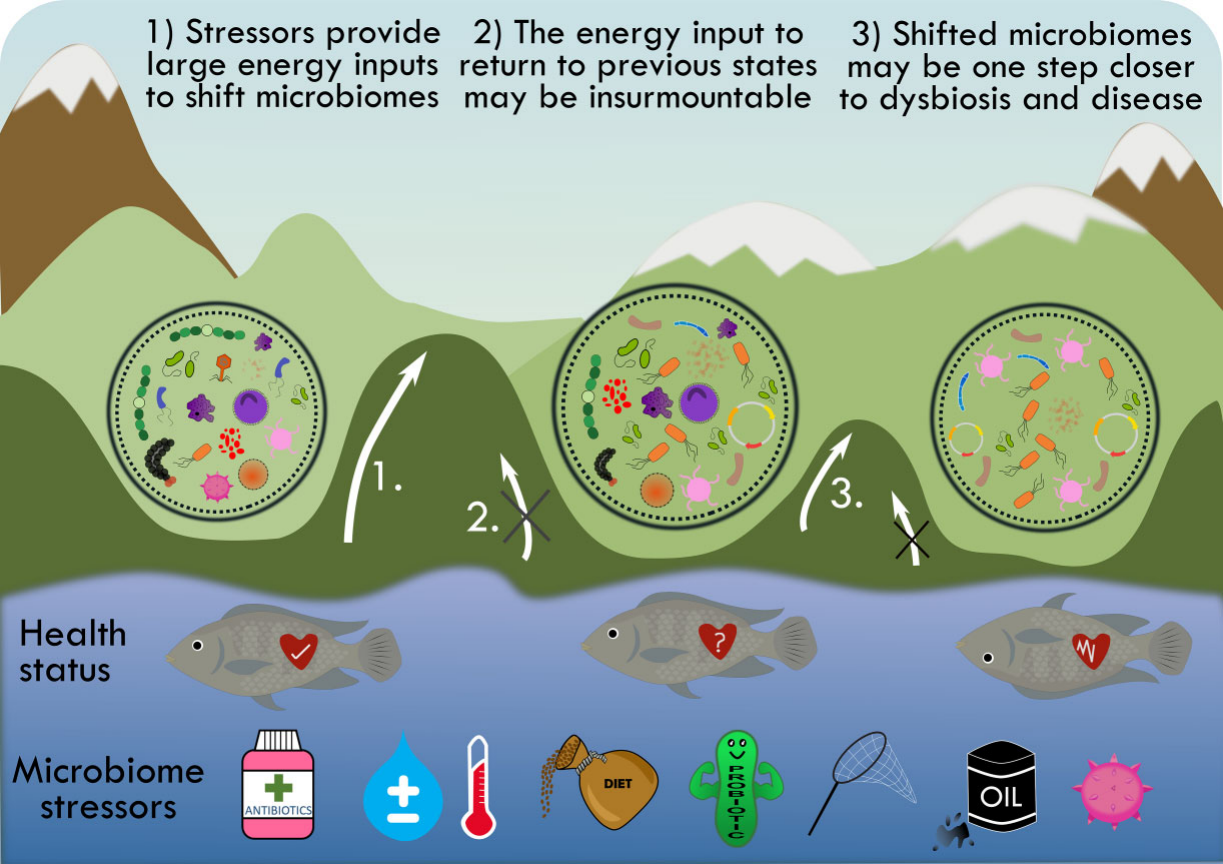
Stress-induced perturbations of fish skin and gill microbiomes. (1) Stressors can shift a microbiome from one stable state to another. (2) In this new state, microbial composition changes, often with an increase in pathobionts and a decrease in commensals, but overall functionality for maintaining health is preserved. This stable state resists reversion due to the high ‘conceptual’ energy required for the shift. (3) Despite functional resilience, altered microbiomes may become more vulnerable to disease, as the ‘conceptual’ energy needed to push the system into dysbiosis is reduced. Subsequent stressors may trigger this transition, leading to disease onset.

Exposure to stressors can induce temporary or permanent dysbiosis in skin and gill microbiomes. In the conceptual ‘energetics landscape’ of a microbiome (Fig. 3), significant perturbations are required to shift a microbiome into a new state and the stability of the microbiome plays a key role in dictating its resilience against perturbation into a dysbiotic state. Dysbiosis, marked by taxonomic shifts favouring pathobionts over commensals, often reduces disease resilience, although the exact relationship with fish health remains unclear. Microbiome plasticity enables communities to maintain functionality despite composition changes (Lorgen-Ritchie et al. 2023) albeit stressors that exceed the natural buffering capacity of microbiomes can disrupt their function (Fig. 3.1) (Lloyd-Price et al. 2016, Levy et al. 2017) leading to permanent shifts (Fig. 3.2). Microbiome health is best assessed by evaluating functional capacity rather than taxonomy (Fig. 3) (Huttenhower et al. 2012, Lloyd-Price et al. 2016, Brumlow et al. 2019), however, without immediate functional changes, altered microbiomes may have increased vulnerability to future stressors if pathobionts expand or commensals are lost (Fig. 3.3).

There are very few studies that have explored the effects of stressors on host health and disease resilience after the induction of a skin or gill microbiome dysbiotic state. In one example, channel catfish (*Ictalurus punctatus*) skin and gill microbiomes were disrupted by the disinfectant potassium permanganate, causing greater susceptibility to *F. columnare* challenge with increased mortality compared to controls, indicating impaired host resilience against this disease (Mohammed and Arias 2015). Another example is mosquitofish (*G. affinis*) with skin microbiome disruption by rifampicin. Subsequently, mosquitofish were exposed to osmotic stress or the pathogen *Edwardsiella ictalurid*, showing increased mortality compared to controls (Carlson et al. 2017). However, in Atlantic salmon with AGD, no increased disease severity was observed in fish treated with oxytetracycline, despite gill microbiome compositional perturbations (Slinger et al. 2021b). This supports the fact that a taxonomically perturbed microbiome may still maintain functionality. However, varying states of perturbation can be induced by microbiome stressors that render the host more susceptible to disease. It is worth noting that stressors can also exert direct impacts on immune function of fish mucosal tissues (Ellison et al. 2018, 2020) and in turn be a potential effector for disruption of microbiome composition. However, the highly interconnected nature of immune and microbiome responses makes it extremely difficult to separate these different effect pathways when considering fish mucosal surface responses to stressors, necessitating a holistic approach.

### Pathobionts in disrupted microbiomes

Pathobionts, typically harmless members of healthy microbiomes, can become opportunistic pathogens in disrupted microbiomes. For example, in rainbow trout, *Staphylococcus warneri* is normally nonpathogenic, but stress can facilitate its expansion and enhance the biofilm formation of the fish pathogen *Vibrio anguillarium* (Musharrafieh et al. 2014). While an increase in pathobionts does not necessarily lead to disease, it can signal a microbiome that is more susceptible to opportunistic infection. Illustrating this, brown and rainbow trout skin injuries were found to harbour ~9000 times more gene copies of the disease-causing oomycete *Saprolegnia parasitica* compared to healthy fish, despite showing no gross pathological signs of disease (Pavić et al. 2022). Such pathobionts enrichment can compromise future health, particularly if further stressors reduce the microbiome’s functional capacity to resist disease.

### Dysbiotic microbiomes and disease states

While diseases are typically attributed to specific pathogen(s), dysbiosis itself can be considered a ‘disease state’, contributing to multifaceted diseases lacking clear etiological agents. For example, white faeces syndrome (WFS) in shrimp (*Penaeus monodon* and *P. vannamei*) has been linked to gut microbiome dysbiosis (Alfiansah et al. 2020, Wang et al. 2020b). WFS-afflicted shrimp exhibit enrichment of *Vibrio, Candidatus Bacilloplasma, Rhodobacter, Chitinbacter*, and *Lactobacillus*, reduced alpha diversity and abnormal microbiome functionality and metabolic activities. It is unclear whether dysbiosis causes or results from WFS, but experiments following Koch’s postulates have helped elucidate the causative relationship. Transplanting dysbiotic microbiota from WFS-affected shrimp into healthy ones induced WFS pathology and repeating this transplantation from newly diseased shrimp into healthy ones also induced WFS development. Conversely, transplanting healthy microbiota reversed WFS pathology, suggesting dysbiosis as the cause of WFS manifestation (Huang et al. 2020). Adopting this approach could both clarify the role of stressor-induced microbiome disruption in disease and help differentiate between microbiome dysbiosis as the cause versus symptom of increased disease susceptibility.

## Future research on fish skin and gill microbiomes

### Expanding our understanding of health impacts

Although there is an increasing body of data on changes that occur in the mucosal microbiomes of fish in response to various stressors, many of these are correlative analyses only. These descriptive changes furthermore allow for inferences only for impacts on fish health. Microbiomes can also differ considerably between individuals and for different environmental contexts, and as such it is challenging to define a healthy microbiome taxonomically. Assessing the functionality of fish skin and gill microbiomes is far better suited for understanding how different microbiome states affect fish health. While studies on fish and human gut microbiomes have made significant progress in understanding microbiome functionality, this level of insight is still lacking for fish skin and gill microbiomes.

Future research needs to include studies into how microbes on the skin and gill prime the host’s immune response, influence inflammation, and increase resilience to pathogens. Describing this ‘cross-talk’ between the microbiome and the immune system, particularly how these interactions develop and maintain healthy skin and gill microbiomes, is essential for identifying mechanisms that reinforce or weaken this protective barrier.

The role of host genetics in shaping microbiome interactions and disease susceptibility is another much-needed research area. Although genetic factors influencing pathogen resistance have been identified in species like Nile tilapia (Barría et al. 2021, Vela-Avitúa et al. 2023), less attention has been paid to how host genetics affects commensals. Identifying host genetics that promote the integration of beneficial microbes into skin and gill microbiomes could inform selective breeding or genetic modification efforts in aquaculture. Such approaches would strengthen the microbiome’s protective role to improve disease resilience.

Another promising avenue of research in fish health treatments is the application of microbiome restoration techniques. Faecal microbiota transplants (FMT) have shown success in restoring fish gut microbiomes and protecting against pathogens in other systems (Legrand et al. 2020, Huang et al. 2020). However, to our knowledge, similar approaches have not been applied to address major problems of fish skin and gill diseases, such as AGD. FMT has successfully treated infections of antimicrobial-resistant *Clostridium difficile* in humans (Liubakka and Vaughn 2016), however, it carries risks, including the introduction of pathogens and antimicrobial-resistant bacteria (Ott et al. 2017). A more targeted approach might identify and cultivate groups of commensal taxa that help restore healthy microbiome functionality on fish skin and gills. Unlike probiotic treatments, these strategies aim to reestablish entire microbial communities, offering more sustainable and effective long-term protection. In addition to disease resistance, restoring skin and gill microbiomes could promote wound healing and tissue regeneration, as some microbial taxa have been shown to aid these processes (Tomic-Canic et al. 2020). Understanding and manipulating beneficial microbes could unlock new therapeutic possibilities, expanding the scope of microbiome research beyond pathogen defence to include broader health and recovery benefits for fish.

### Tools for advancing functional understanding of fish skin and gill microbiomes

Research on fish skin and gill microbiomes has predominantly focused on microbial diversity and composition using 16S rRNA metabarcoding, but this approach lacks insight into microbiome functionality. Methods to bioinformatically predict function from short hypervariable fragments of the 16S rRNA gene are questionable (Heidrich and Beule 2022), particularly in environmental systems as functional assumptions are largely drawn from human studies (Sun et al. 2020). To bridge this gap, metagenomics and metatranscriptomics provide more reliable functional predictions for characterizing the metabolic pathways within (fish skin and gill) microbiomes. Metagenomics allows for the identification of genes involved in, for example, nutrient cycling, biofilm formation, or antimicrobial resistance (Carda-Diéguez et al. 2017, Bell et al. 2023). Metatranscriptomics provides dynamic insights into the active metabolic pathways of the microbiome and can show how microbial communities actively respond to stressors like pollutants, infections, or environmental changes. These methods can provide a comprehensive understanding of microbial capabilities, but high host DNA content in fish skin and gill samples hampers microbial sequence recovery. Using host DNA depletion techniques during extraction or sequencing (Heravi et al. 2020, Loose et al. 2016) can enrich the output of microbial sequencing data to increase the fraction of microbial genes recovered in skin and gill samples. Additionally, to avoid host DNA, specific genes and pathways of interest can be targeted by quantitative Polymerase Chain Reaction (qPCR)/ digital droplet Polymerase Chain Reaction (ddPCR). This refines functional profiling by allowing direct comparisons of functional markers of the microbiome (Crane et al. 2018). Thus, offering complementary insights into microbiome stressor responses when combined with traditional metabarcoding approaches.

Metabolomics complements these genomic tools identifying the actual metabolic products of microbiomes, offering direct evidence of microbiome activity. For example, in gills of parasitized butterflyfish (*Chateodon lunulatus*), shifts in metabolomics profiles have been linked to specific changes in microbial taxa (Reverter et al. 2020), suggesting that microbial communities may influence host metabolic pathways that are critical for maintaining tissue health or combatting infections. Tracking these shifts offers a real-time assessment of how microbiomes functionally respond to changes in the environment or host health.

Single-cell genomics combined with flow cytometry offers the ability to isolate and sequence individual microbial cells, even for those present at low abundance (Madhu et al. 2023). This method allows for the detailed study of rare but potentially critical microbial taxa, such as those involved in skin healing or immune modulation. By excluding host cells during isolation, single-cell genomics can provide high-resolution functional profiles of microbiomes, helping to identify microbial genes responsible for antiinflammatory functions, wound repair, or resistance to external pathogens (Lloréns-Rico et al. 2022). However, to date, this technology has not been applied to gain a functional understanding of fish microbiomes.

Finally, *in vitro* model systems such as synthetic fish skin with engineered microbial communities present a tractable tool for studying microbiome colonization, biofilm formation, and interactions with environmental stressors in a controlled environment. These synthetic models have been developed to simulate human skin (Lekbua et al. 2024) and Atlantic salmon gut microbiomes to assess the microbiome impacts of prebiotic treatments (Kazlauskaite et al. 2021, 2022). If developed to simulate fish skin or gill mucosal microbiomes, researchers could manipulate stressors to observe functional microbial responses, while removing the variability and ethical issues of live fish experimental systems.

### Moving beyond the single-stressor paradigm

Most studies reviewed have experimentally applied a single stressor, often overlooking other contributing factors. However, stressors rarely occur in isolation and are generally interactive, potentially additive, or even synergistic in their effects on system resilience. Illustrating this in barramundi (*Lates calcarifer*), together cold water stress, mechanical skin wounding, and pathogenic challenge by *V. harveyi* caused increased mortality rates when applied cumulatively (Samsing et al. 2023). Assessing the interactive effects of multiple environmentally relevant stressors on microbiomes that underpin health will become increasingly important in the face of climate change, as fish will increasingly experience environmental conditions outside their normal physiological ranges, both in the wild and in aquaculture.

### Use of microbiomes and microbial biomarkers in health management

Some studies have identified microbiome biomarkers, such as the proliferation of pathobionts or elimination of commensals, to signify changing health outcomes or disease onset in fish (Mougin and Joyce 2023). For example, the bacterial species *Mycoplasma* shows proven host-commensal coevolution patterns in Atlantic salmon gut microbiomes (Rasmussen et al. 2023). *Mycoplasma* abundance increases with diet supplementation with prebiotics (Baumgärtner et al. 2022) but decreases with parasitic gut cestode (Brealey et al. 2022) and bacterial skin infection (Bozzi et al. 2021). Alternatively, pathobionts act as negative biomarkers for health. However, pathobionts are often inappropriately categorized according to taxonomic similarity to known pathogens, typically at the genus level which can include commensal microorganisms (Jochum and Stecher 2020). For example, many species and strains within a genus such as *Pseudomonas* have proven probiotic and mutualistic properties but also include pathogenic taxa (Ringø et al. 2022).

Biomarker identification (of both commensals and pathobionts) is context-dependent, with interindividual variation occurring for both fish microbiomes and stressor responses. Future research needs to coordinate efforts to identify biomarkers, potentially through meta-analyses (Bell et al. 2024) that identify conserved microbiome responses to stressors that contribute towards adverse health outcomes. Biomarker responses can then be assessed through longitudinal studies during disease events or stressor exposure. Knock-out experiments might be employed to elucidate the functional health contributions played by specific commensals. Once functional importance is confirmed within a defined microbiome, such as *Mycoplasma* in the gut of Atlantic salmon, using specific biomarker taxa in monitoring should provide a valuable tool to assess fish health and disease progression, in both wild fish and aquaculture settings.

## Concluding remarks

Physical, chemical, and biological stressors cause diverse and multifaceted disruptions to fish skin and gill microbiomes, generally resulting in shifts of microbial diversity, the proliferation of pathobionts, and the depletion of commensals. However, functional redundancy ensures microbiome resilience, allowing a system to resist dysbiosis and maintain host health even with changes in microbial composition. In turn, this emphasizes the crucial need to understand alterations that lead to disruptions of microbiome function. A better understanding of the functional redundancy of these microbiomes is an important element in these function-directed studies and in determining their resilience to disruption. Skin and gill microbiomes are dynamic entities, exhibiting a very wide range of different states, and no single profile defines a discrete state of health or disease. Emerging evidence indicates that cumulative stressors, rather than single events, disrupt these functional states, leading to disease. However, most studies use correlational data, making causation unclear. Future research should isolate specific mechanisms linking stressors to microbiome disruption and disease. Understanding the interplay between functional redundancy and microbiome resilience is essential for mitigating disease in aquaculture while supporting resilient fish populations and ecosystem stability.
